# Supplementary material for: Motor response vigour and visual fixation patterns reflect subjective valuation during intertemporal choice
Source: PLoS Comput Biol. 2022 Jun 10;18(6):e1010096. doi: 10.1371/journal.pcbi.1010096 (PMC9187114; doi:10.1371/journal.pcbi.1010096)
Supplement: S1 Text — Fig A: Posterior predictive response time distributions of the DDMsig-shift (using absolute values) for each participant, overlaid on the histograms of the observed RT distributions. Fig B: Parameters of the modelled grip response (mean values per participant and magnitude condition). Fig C: Within-subject differences of the parameters of the modelled grip response between the low and high magnitude condition. Fig D: Hierarchical Bayesian regression of the parameters of the Gaussian-modelled grip force response and number of fixation shifts onto the trial-wise response conflict based on the drift rate (DDM). Fig E: Hierarchical Bayesian regression of the parameters of the Gaussian-modelled grip force response and number of fixation shifts onto the subjective value differences (DDM). Fig F: Hierarchical Bayesian regression of the parameters of the Gaussian-modelled grip force response and number of fixation shifts onto the total sum of the option amounts (model-free). Fig G: Hierarchical Bayesian regression of the parameters of the Gaussian-modelled grip force response and number of fixation shifts onto the total sum of the subjective option values from the drift diffusion model (DDM) for the low magnitude condition. Fig H: Hierarchical Bayesian regression of the parameters of the Gaussian-modelled grip force response and number of fixation shifts onto the total sum of the subjective option values from the drift diffusion model (DDM) for the high magnitude condition. Section 3: Conflict based on trial-wise drift rate (DDM). Section 4: Conflict based on subjective value differences (DDM using normalised values). Section 5: Sum of larger-later (LL) and smaller-sooner (SS) amounts (model-free). Section 6: Sum of the subjective larger-later (LL) and smaller-sooner (SS) option values (DDM) per magnitude condition. (PDF) [file pcbi.1010096.s001.pdf]

Supplementary material

Motor response vigour and visual fixation patterns reflect subjective valuation during intertemporal choice

Elke Smith[1*], Jan Peters[1]

1 Department of Psychology, Biological Psychology, University of Cologne, Cologne, Germany

*e.smith@uni-koeln.de

1 Posterior predictive response time distributions

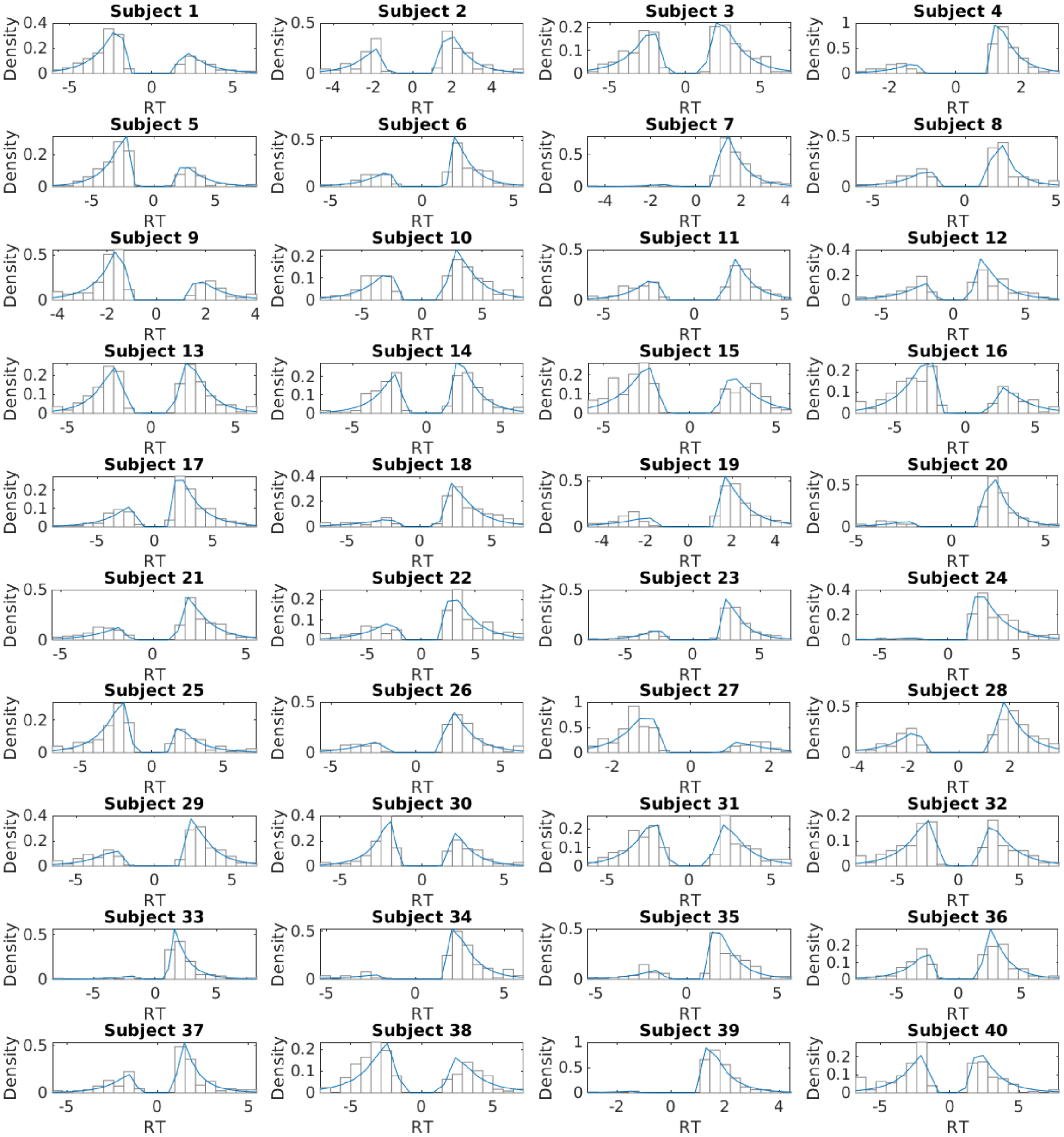

Fig A. Posterior predictive response time distributions of the $DDM_{\text{sig-shift}}$ (using absolute values) for each participant, overlaid on the histograms of the observed RT distributions. The negative response times arise from the boundary definitions of the DDM. We defined the lower boundary as choosing the SS reward, and the upper boundary as choosing the LL reward. For this purpose, choices towards the lower boundary were multiplied by -1. Negative response times indicate SS choices, positive response times indicate LL choices.

2 Gaussian grip force model

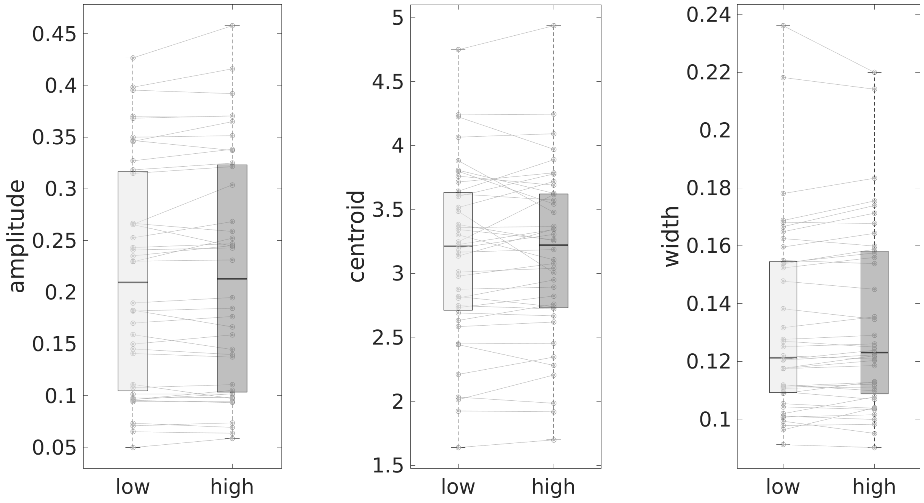

Fig B. Parameters of the modelled grip response (mean values per participant and magnitude condition). The handgrip response was modelled with a Gaussian function with 1 term plus a constant (see Eq 10). Amplitude has been normalised to MVC (maximal voluntary contraction). Centroid and width are reported in seconds.

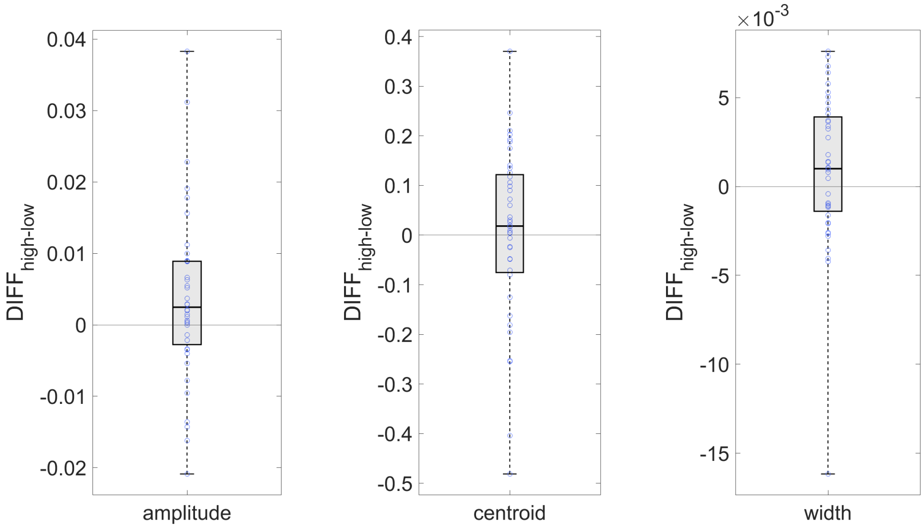

Fig C. Within-subject differences of the parameters of the modelled grip response between the *low* and *high* magnitude condition. The handgrip response was modelled with a Gaussian function with 1 term plus a constant (see Eq 10). Amplitude has been normalised to MVC (maximal voluntary contraction). Centroid and width are reported in seconds.

3 Conflict based on trial-wise drift rate (DDM)

Response conflict was also operationalised based on the trial-wise drift rate calculated based on the estimated parameters of the highest-ranked DDM using normalised values ($DDM_{sig\text{-}shift}$). The posterior distributions of the group-level parameter means for the regression coefficients are depicted in Fig D (medians: α = 0.29 [intercept] β_1 = 0.03 [amplitude], β_2 = -0.02 [centroid], β_3 = -0.04 [width], β_4 = -0.05 [N fixation shifts]). The Bayes factors provide only anecdotal evidence that the regression coefficient for grip force amplitude is greater than zero vs. smaller than zero (BF for β_1: 1.29), or that the coefficients for centroid, width and number of fixation shifts are below zero rather than above zero (BF for β_2: 1.28, BF for β_3: 1.57, BF for β_4: 1.72). Since for all βs, the 95% HDIs of the posterior distribution lie neither completely inside nor outside the ROPE, we remain undecided for these regression coefficients.

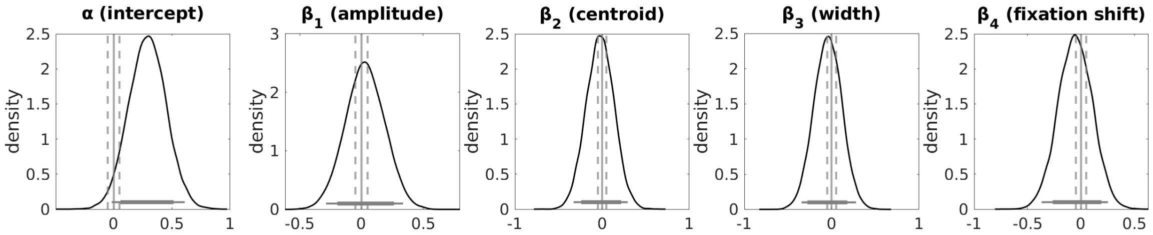

Fig D. Hierarchical Bayesian regression of the parameters of the Gaussian-modelled grip force response and number of fixation shifts onto the trial-wise response conflict based on the drift rate (DDM). Posterior distributions of the group-level parameter means. α: intercept, $\beta 1$: coefficient for amplitude, β_2: coefficient for centroid, β_3: coefficient for width, β_4: coefficient for fixation shift. Horizontal solid lines indicate the 85% and 95% highest density interval. Vertical solid lines indicate x = 0, and vertical dashed lines indicate the lower and upper bounds of the region of practical equivalence (ROPE).

4 Conflict based on subjective value differences (DDM using normalised values)

The medians of the group-level posterior distributions were as follows: α = 0.16 (intercept) β_1 = 0.01 (amplitude), β_2 = -0.02 (centroid), β_3 = 0.001 (width), β_4 = -0.004 (N fixation shifts). The Bayes factors for all β regression coefficients (for amplitude, centroid, and width of the grip response and number of fixation shifts) provide only anecdotal evidence for values greater than zero (BF for β_1: 1.07, BF for β_2: 0.78, BF for β_3: 1.02, BF for β_4: 0.89). We remain undecided for all β coefficients, since the 95% HDI of the posterior distribution is neither completely inside nor outside the ROPE (see Fig E).

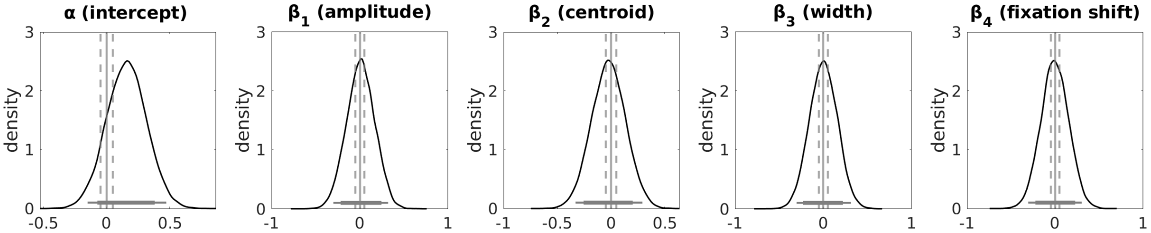

Fig E. Hierarchical Bayesian regression of the parameters of the Gaussian-modelled grip force response and number of fixation shifts onto the subjective value differences (DDM). Posterior distributions of the group-level parameter means. α: intercept, $\beta 1$: coefficient for grip force amplitude, β_2: coefficient for grip force centroid, β_3: coefficient for grip force width, β_4: coefficient for fixation shift. Horizontal solid lines indicate the 85% and 95% highest density interval. Vertical solid lines indicate x = 0, and vertical dashed lines indicate the lower and upper bounds of the region of practical equivalence (ROPE).

5 Sum of larger-later (LL) and smaller-sooner (SS) amounts (model-free)

To analyse the association between motor response vigour, number of fixation shifts and total value, we regressed the parameters of the Gaussian grip force model and the number of fixation shifts onto the sum of the LL and SS option amounts. The medians of the group-level posterior distributions were as follows (see Fig F): α = 0.51 (intercept), β_1 = 0.53 (amplitude), β_2 = 0.19 (centroid), β_3 = 0.19 (width), β_4 = -1.29 (N fixation shifts). The Bayes factor for the regression coefficient for peak force provides very strong evidence for values greater than zero vs. smaller than zero (BF for $\beta 1$: 41.07). The Bayes factor for the coefficients for centroid and width provide only anecdotal and moderate evidence, respectively, for values greater than zero vs. smaller than zero (BF for $\beta 2$: 2.79, BF for $\beta 3$: 3.04). The Bayes factor for the coefficient for number of fixation shifts provides extreme evidence for values smaller vs. greater than zero (BF for $\beta 4$: $> 10^{308}$). For $\beta 1$, $\beta 2$ and $\beta 3$ we remain undecided, since the 95% HDI of the posterior distribution is neither completely inside nor outside the ROPE. For $\beta 4$ we reject the null value (95% HDI of posterior distribution entirely outside ROPE).

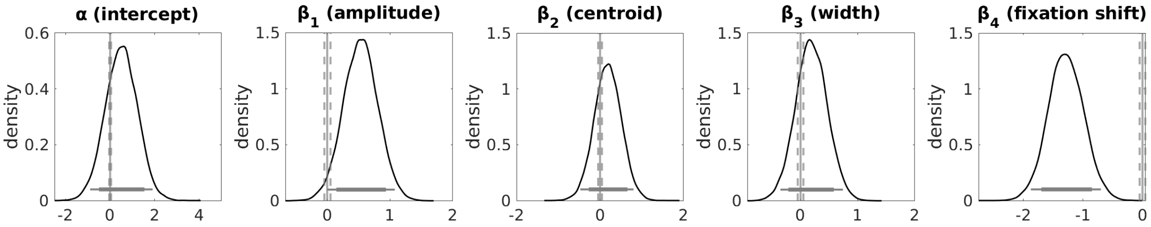

Fig F. Hierarchical Bayesian regression of the parameters of the Gaussian-modelled grip force response and number of fixation shifts onto the total sum of the option amounts (model-free). Posterior distributions of the group-level parameter means. α: intercept, β_1: coefficient for grip force amplitude, β_2: coefficient for grip force centroid, β_3: coefficient for grip force width, β_4: coefficient for fixation shift. Horizontal solid lines indicate the 85% and 95% highest density interval. Vertical solid lines indicate x = 0, and vertical dashed lines indicate the lower and upper bounds of the region of practical equivalence (ROPE).

6 Sum of the subjective larger-later (LL) and smaller-sooner (SS) option values (DDM) per magnitude condition

6.1 *Low* magnitude condition

The medians of the group-level posterior distributions were as follows: α = 0.94 (intercept), β_1 = 0.12 (amplitude), β_2 = -0.30 (centroid), β_3 = -0.19 (width), β_4 = -0.56 (N fixation shifts) (see Fig G). The Bayes factor for the regression coefficient for grip force amplitude provides anecdotal evidence for values greater than zero vs. smaller than zero (BF for β_1: 2.81). The Bayes factor for the regression coefficient for the grip centroid provides strong evidence for values smaller than zero vs. greater than zero (BF for β_2: 10.40), the Bayes factor for the regression coefficient for the width parameter provides moderate evidence for values smaller than zero vs. greater than zero (BF for β_3: 4.73), and the Bayes factor for the regression coefficient for the number of fixation shifts provides extreme evidence for values smaller than zero vs. greater than zero (BF for β_4: 184.48). Since the 95% HDIs of the posterior distributions for β_1, β_2, and β_3 fall neither completely inside nor outside the ROPE, we remain undecided for these three regression coefficients. For β_4 we reject the null value (95% HDI of posterior distribution entirely outside ROPE).

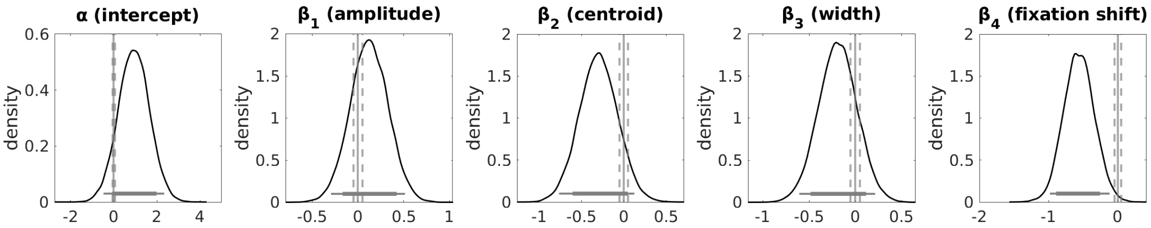

Fig G. Hierarchical Bayesian regression of the parameters of the Gaussian-modelled grip force response and number of fixation shifts onto the total sum of the subjective option values from the drift diffusion model (DDM) for the *low* magnitude condition. Posterior distributions of the group-level parameter means. α: intercept, β_1: coefficient for grip force amplitude, β_2: coefficient for grip force centroid, β_3: coefficient for grip force width, β_4: coefficient for fixation shift. Horizontal solid lines indicate the 85% and 95% highest density interval. Vertical solid lines indicate x = 0, and vertical dashed lines indicate the lower and upper bounds of the region of practical equivalence (ROPE).

6.2 *High* magnitude condition

The medians of the group-level posterior distributions were as follows: α = 0.99 (intercept), β_1 = 0.53 (amplitude), β_2 = -1.18 (centroid), β_3 = 0.11 (width), β_4 = -0.83 (N fixation shifts) (see Fig H). The Bayes factor for the regression coefficient for grip force amplitude provides very strong evidence for values greater than zero vs. smaller than zero (BF for β_1: 60.27). The Bayes factor for the regression coefficient for the grip centroid provides extreme evidence for values smaller than zero vs. greater than zero (BF for β_2: 2181.35), the Bayes factor for the regression coefficient for the width parameter provides moderate evidence for values greater than zero vs. smaller than zero (BF for β_3: 2.19), and the Bayes factor for the regression coefficient for the number of fixation shifts provides extreme evidence for values smaller than zero vs. greater than zero (BF for β_4: 593.96). For β_2 and β_4 we reject the null value (95% HDI of posterior distribution entirely outside ROPE). For β_1 we also reject the null value, since the 95% HDI dos not include zero and only 0.63% of the 95% HDI overlaps with the ROPE. For β_3 we remain undecided, since the 95% HDI of the posterior distribution is neither completely inside nor outside the ROPE.

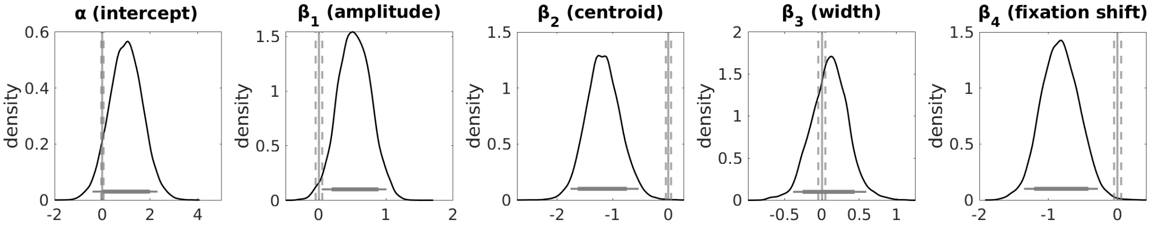

Fig H. Hierarchical Bayesian regression of the parameters of the Gaussian-modelled grip force response and number of fixation shifts onto the total sum of the subjective option values from the drift diffusion model (DDM) for the *high* magnitude condition. Posterior distributions of the group-level parameter means. α: intercept, β_1: coefficient for grip force amplitude, β_2: coefficient for grip force centroid, β_3: coefficient for grip force width, β_4: coefficient for fixation shift. Horizontal solid lines indicate the 85% and 95% highest density interval. Vertical solid lines indicate x = 0, and vertical dashed lines indicate the lower and upper bounds of the region of practical equivalence (ROPE).
