# Supplementary material for: Motor response vigour and visual fixation patterns reflect subjective valuation during intertemporal choice
Source: PLoS Comput Biol. 2022 Jun 10;18(6):e1010096. doi: 10.1371/journal.pcbi.1010096 (PMC9187114; doi:10.1371/journal.pcbi.1010096)
Supplement: Attachment — Submitted filename: Response_to_reviewers.pdf [file pcbi.1010096.s004.pdf]

Reviewer #1

Smith and Peters investigate the potential of response vigour (through gaze shifting and grip force) as an implicit measure of subjective utility during response selection of during intertemporal-choice based decision making. By combining psychophysics and modellings, the authors report a utility of response vigour in examining different aspects of decision making.

The strengths of this manuscript are two-fold. First, the authors leverage a well-thought-out and well-controlled psychophysical paradigm with rigorous cognitive models and statistical analyses, which, together, provide important findings regarding the nature of decision making. Second, the reported observations provide foundation for future experimental and computational work investigating various aspects of perceptual information processing and decision making both in health and disease.

Additionally, I appreciate the clarity with which the authors describe each component of the modelling and statistical analyses. I believe the authors' choices for these methods are well-justified and appropriate for the question investigated. Below I have a few comments and questions that I hope the authors could discuss as they could further help place the reported findings in context of past theoretical and empirical studies.

1. The authors excluded trials where the responses were made either 'too early' or 'too late'. How were the 200ms and 10s criteria were chosen? Can we think of these as 'false alarms' and 'missed' trials, accordingly? While the total number of trials that fell into these categories were small (1.81% of trials), I strongly believe it is an important conceptual idea to be considered/discussed. Specifically, the number of these trials heavily dependent on task difficulty (e.g., differential payoff/reward across conditions, time pressure), and I'm wondering if the authors could discuss how the number of 'too early' and 'too late' responses as well as task difficulty can be incorporated and interpreted in the context of the reported modelling results.

    > Thanks, this raises an important issue. Valid response times are physiologically limited to a lower bound of around 100 to 200 ms, arising from the need for stimulus perception and encoding and motor response execution (Berger & Kiefer, 2021; Luce, 1991; Whelan, 2008). Very fast response times usually do not reflect the process one is interested in, and rather result from premature responding or blind guessing. Based on these considerations, we excluded trials with response times below 200 ms. The upper response time cut-off derives from the task instruction. The participants were instructed that there was no time pressure for the decision, but that they should decide according to their gut feeling and not think too long about the decision. Since the task was comparatively simple, reaction times > 10 s are likewise rather implausible and likely reflect a lack of attention rather than the process of interest.
    > The term false alarm originates from signal detection theory (SDT). It refers to the incorrect categorisation of a stimulus as belonging to the target category. Therefore, in an SDT sense, fast response times do not directly correspond to false alarms, since there is no defined target category in our experiment. Misses usually either refer to trials with no response at all, or, following SDT terminology, trials in which the stimulus belonged to the target category, but was

not classified as such. In the former sense, we would consider trials with very long response times (> 10s) as missed trials, since these trials likely reflect lapses of attention. We now include these considerations regarding response time cut-offs in the manuscript (section 2.5.1), where we now write:

“Since valid response times are physiologically limited to a lower bound of around 100 to 200 ms [32, 33], we excluded trials with response times below 200 ms. Further, we excluded trials with response times > 10 s. The participants were instructed that there was no time pressure for the decision, but that they should decide according to their gut feeling and not think long about the decision. Since the task was comparatively simple, long reaction times likely reflect a lack of attention rather than the process of interest. Finally, we excluded trials with maximum grip force values falling below the threshold for logging a response (technical issue with faulty signal on parallel port). In total, 139 trials (1.81% of trials) from 26 participants were excluded.”

2. Along the line of the previous comment, I believe the interpretation of non-decision time (t0) is also dependent of the task setup and temporal criteria for what trials/responses to be excluded from the analyses. Can the authors expand more on their discussion of t0 in light of this?

> We agree with the reviewer. For example, we observed considerably longer non-decision times in the present study, compared to some of our previous work. This discrepancy is, as suggested by the Reviewer, likely attributable to the specific task setup. For example, the setup included the requirement of first preselecting an option by fixation and pressing a force transducer instead of a regular response key. We expand on this in the discussion (see section 4). The non-decision time parameter τ models time that is not related to the decision process, such as stimulus encoding and motor response execution. However, τ does not take into account physiological plausibility. In particular, if all trials were included, the non-decision time would necessarily adjust, shifting the modeled RT distribution towards zero as much as required to ensure a probability density > 0 for fast outlier trials. As noted above, response times < 200 ms are considered physiologically implausible, such that exclusion is warranted to ensure that such implausible responses do not exert an undue influence on the modeled RT distributions.

3. I firmly believe that the findings reported in this manuscript have far reaching implications across many fields including experimental psychology, cognitive/clinical neuroscience, and computational psychiatry (as the abstract, author summary, and introduction alluded to). Yet, the discussion does not include sufficient text and references to highlight these points. One important area where I think the reported findings could be very beneficial is studies of information processing and decision making in amnesic patients as well as those aiming to examine conscious/unconscious processing related to decision making. For example, there exist several studies that reported that amnesic patients are impaired in certain processes such as statistical-learning based decision making using explicit reports (Schapiro, Gregory, Landau, McCloskey, Turk-Browne, J Cog Neuro, 2014). However, patients were found to be intact on these tasks when tested using implicit reports (e.g.,

Rungratsameetaweemana, Squire, Serence, PNAS, 2019). Along this line, I think the reported findings regarding the use of response vigour could greatly benefit future investigations in various clinical populations where explicit reports may not be possible/ reliable.

> Thanks for raising this issue. We now address these points in the discussion, where we now write:

"Using response vigour as an implicit measure of utility may open up the possibility to assess utility in cases where explicit reports are not possible, i.e. in different patient groups. In the area of statistical learning, patients with hippocampal damage show impairments in certain processes when the patients are required to explicitly report regularities or patterns [64, 65]. When testing for implicit knowledge in a motion discrimination task, patients with hippocampus damage showed a similar performance to controls [66]. Experimental approaches such as those employed in the present study might be informative in such patient populations. Furthermore, there is evidence that effort processing is impaired in individuals with disorders characterised by motivational deficits such as depression, schizophrenia and Parkinson's disease [14, 67, 68]. Since motivational deficits are hallmarks of various psychiatric disorders [69], implicit utility measures such as response vigour may be informative with respect to the motivational deficits in such patient populations."

4. Are the three sample trials for Fig 3 picked at random and are they, by and large, representative of 1) the overall modelled grip response of this subject on other trials and 2) averaged response across participants?

> The three sample trials depicted in Fig. 3 (section 3.4) are three random trials from one participant (not averaged across participants or trials). They are representative for most of the other modelled grip responses. This is also evident from the fact that the R^2 values for these trials were 0.983, 0.998 and 0.997, which is similar to the overall goodness of fit of the response model across trials and subjects (mean R^2 = 0.98). We added further information to the figure caption (see also next point).

5. I feel that the captions of most figures could use additional explanation. For example, in Figure 3, the authors may consider labeling each of the three panels as panel a, b, c, and include text in the caption to describe what's being plotted in blue and black etc. The tables could also benefit from more complete labels. For example, in table 4, the definitions of alpha, z, etc. are missing.

> Following the reviewers suggestion, we added further information to the figure caption of Figure 3, to make it more clear what is being displayed. Also, we labelled the three sub-panels (a-c) (section 3.4).

> We added descriptions of the parameters listed in Table 4 and 5 (section 3.3.3) to the table notes. We carefully checked the other tables and figures and added further information where necessary (e.g. Table 1, section 3.2).

6. I would like to highlight that the authors did a great job walking readers through each of the modelling components. The modelling results were also clearly explained. However, it is unclear why the softmax rule models were models of choice in the context this task. While the DDM was well motivated in the introduction, some more background concerning why the softmax rule models were appropriate and of interested (and better candidates than other models not considered) will be very helpful.

    > We thank the reviewer for pointing out that we have to provide further information about the choice of models (section 2.4.2):

    “The softmax action selection rule is a commonly used choice rule for value-based decision making and reinforcement learning, applied in our own and others’ previous work [36, 37, 26]. Here we applied this model as a baseline or reference model.”

Reviewer #2

The manuscript reports the results of a pre-registered study that investigated how implicit behavioral measures (gaze fixations and grip force) are affected by the subjective values of options presented during an intertemporal choice task. Of interest, there were links with the overall value of the option pair and the difference between option values.

Among the strengths of the paper is an original choice task in which options are selected by visual fixation and confirmed by pressing a handgrip. Another is the sophistication of the computational analyses and the use of Bayesian techniques for model selection. My global impression however is that the paper lacks focus and fails to identify the main questions of interest. As stated in my summary above, the analyses should target the links between the two implicit measures (visual fixations and grip force) and the two computational variables (value sum and difference). This could be done in a model-free analysis that would be straight and simple. In the current paper, these analyses are buried within tons of computational variants that address no meaningful issue at the conceptual level. For instance, whether the DDM provides a better account of RT when taking as input an affine or a sigmoid transformation of value difference will not change our understanding of how the brain works. My main suggestion would be to streamline the analyses around the questions that might bring some conceptual advance. I understand there is the issue of pre-registration, which constrains the authors to sticking on their first ideas. While I understand the virtue of pre-registration in the context of a replication study (where one wants protection against false positives), I think it is problematic in the context of an exploration study (where one wants protection against false negatives) that examines novel potential effects, as we have here. I would therefore encourage the authors to go beyond their pre-registered analyses (which could be moved in supplementary material) and explore their data in a more systematic approach.

More specific concerns:

1) The introduction does not provide a justification for the design. If the idea is to test the link between response vigor and subjective value, then why using intertemporal choice, and why manipulating reward so as to induce a magnitude effect? It also introduces useless or misleading concepts, such as effort discounting, that are never used later in the paper.

> Thanks for raising this issue. The rationale for addressing this research question using intertemporal choice was threefold, and we have now rewritten parts of the introduction (see section 1) to make this clearer. Here we point out that, first, there are well-established models to describe subjective valuation during intertemporal choice, allowing for a well-grounded modelling of the relationship between response vigour and subjective utility. Of course, other tasks involving subjective valuation of options would be conceivable as well, and we now emphasise this in the discussion (see section 4). Second, and more importantly, the magnitude effect for temporal

discounting is a well-replicated behavioural effect in the temporal discounting literature. This allowed us to directly test the hypothesis that this effect would also be reflected at the level of response vigour. Third, alterations in temporal discounting have been linked to a range of psychiatric conditions (Karakula et al., 2016, *Drug Alcohol Depend*; Moreira et al., 2019, *Eur Psychol*, Yi et al., 2010), making this task and construct particularly interesting from a clinical perspective.

> Thanks for raising the question of whether "effort discounting" deserves to be mentioned in the introduction. We believe it is an important concept to consider in the context of the present study. When studying the relationship between subjective valuation and grip force, it is relevant to point out that physical effort, when required by the task, is also considered a response cost, which gives rise to the phenomenon of effort discounting. It is precisely against this background that our results provide additional insight. Although effort is considered a response cost in the effort discounting literature, in the present study, higher effort was nevertheless expended for rewards with higher subjective values, even though effort was not task-relevant. This implies that, in addition to reflecting a response cost , effort also reflects motivational processes. We now address this issue in greater detail in the discussion (section 4), where we now emphasise:

"In contrast to Pessiglione and colleagues [17], where the force produced was related to the payout (reward height magnitude was presented subliminally), we kept the force produced unrelated to the payout. Therefore, even when the force produced is unrelated to the payout (and the participants are unaware that force production is being measured), it is nonetheless related to the subjective value difference and even more so, the value sum. This finding also has implications for the larger literature on effort discounting. Here, physical effort, similar to delay, is considered a decision cost [3, 4]. In contrast, in the present study, the participants applied more force in trials with higher value differences, and in particular a higher subjective value sum of the options. This suggests that motivational processes are also reflected in motor response vigour."

2) Among the four predictions, only the second and third ones (ii and iii) relate to the main question (correlates of value sum and difference). The first prediction, as it is phrased, is trivially false (participants do not always prefer smaller-sooner over larger-later rewards, it obviously depends on rewards and delays). The fourth prediction about the magnitude effect could be reduced to the second (whether value sum affects response vigor). The questions about whether delay and reward magnitude have an influence on choices, which are conflated with predictions (i) and (iv) could be side-tracked as already answered in many previous studies.

> Thanks for pointing this out. Following the reviewer's suggestions, we have clarified the phrasing of the hypotheses in section 1. Note, however, that hypotheses 2 and 4 are not redundant, since 2 refers to trial-wise values, and 4 refers to the magnitude condition:

(i) “Delay influences reward evaluation: Participants show a tendency to devaluate rewards that are temporally distant (temporal discounting).

(ii) Subjective utility modulates response times and grip force: Faster response times and stronger effort (handgrip force) for choices with high utility (subjective value).

(iii) Decision conflict is reflected in motor response vigour and visual fixation patterns: Longer deliberation (response time), less vigour (grip force) and more frequent fixation shifts between the options during high-conflict decisions (choice options with similar subjective value).

(iv) Higher rewards are discounted less and elicit more effort: Lower discount rates, faster response times, and greater motor response vigour (grip force) for larger rewards (between-conditions magnitude effect).”

3) There are fluctuations in the notion of response vigor, which designates grip force specifically in some instances (such as in the abstract and title) and all non-instrumental measures (including number of fixations) in other instances (such as in the first sentence of the conclusion).

> We thank the reviewer for pointing out that we have used the terminology inconsistently. Accordingly, we have made it more clear when we specifically mean motor response vigour (grip force trajectory) and fixation shifts, respectively.

4) I have nothing against using DDM to predict RT, but in the context of this paper it seems like an unnecessary complication. The proxy for confidence (or inverse conflict), i.e. value difference, could be taken directly from the softmax function. Similarly, fitting a response function with 3 parameters to force pulses seems like a good idea, but probably weakens the analyses testing the link between grip force and value-related variables. This is because these correlated parameters compete to explain the same variable in the multiple regression model. I suggest using a model-free measure (like the area under the curve) for a more sensitive and straightforward analysis.

> Thanks for raising this point. Regarding the first point (softmax): Yes, a similar analysis could have been computed using a softmax model. However, this would entail that information contained in the response times is not taken into account. We (Peters & D’Esposito, 2020; Wagner et al., 2020) and others (Ballard & McClure, 2019; Fontanesi et al., 2019; Miletic et al., 2020; Shahar et al., 2019) have argued that the inclusion of response times in computational models of value-based decision-making can yield more sensitive, reliable and informative parameter estimates.

> Regarding the second point (model complexity issue due to the 3-parameter gaussian model): We addressed this issue and carried out the suggested analysis. In short, replacing the 3-parameter gaussian model with a single measure (AUC) yielded no substantial effects, suggesting that the gaussian model is more sensitive, and/or jointly accounting for all aspects of the grip force trajectory (amplitude, width and centroid) increases the sensitivity to detect associations with subjective value.

> In detail, we computed the area under the curve (AUC) for each trial via the approximate integral of the measured trial-wise force trajectory by means of trapezoidal integration using MATLAB’s trapz function. We then regressed AUC and the number of fixation shifts onto the subjective value differences (that is, we replaced the parameters of the Gaussian grip force model by AUC). The results can be seen in Response Letter Figure 1.

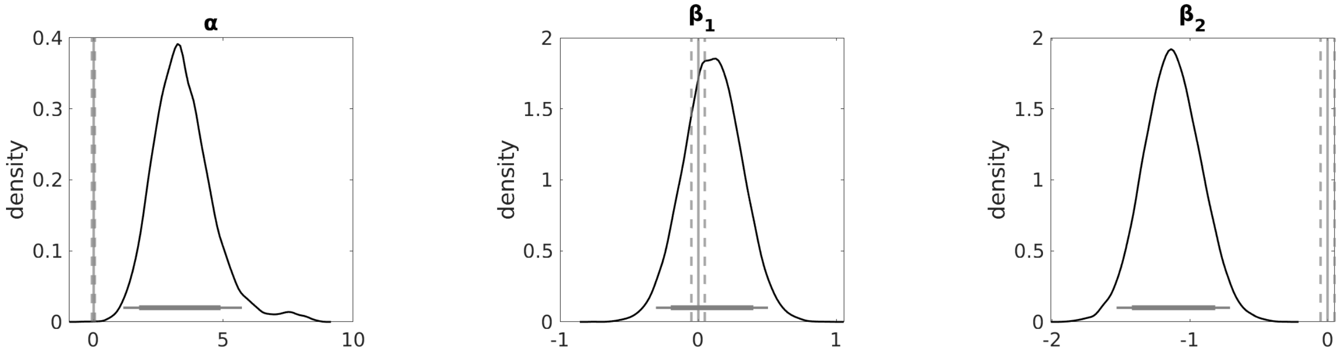

Figure 1. Hierarchical Bayesian regression results. Regression of area under the curve (AUC) and number of fixation shifts onto the value differences (DDM). Posterior distributions of the group-level parameter means. α: intercept, β_1: coefficient for AUC, β_2: coefficient for fixation shift. Horizontal solid lines indicate the 85% and 95% highest density interval. Vertical solid lines indicate x = 0, and vertical dashed lines indicate the lower and upper bounds of the region of practical equivalence (ROPE).

> The Bayes factor for the regression coefficient for AUC provides only anecdotal evidence for values greater than zero vs. smaller than zero (BF for β_1: 2.27). The Bayes factor for the regression coefficient for the number of fixation shifts provides extreme evidence for values smaller than zero vs. greater than zero (BF for β_2: > 10^{308}). Looking at the ROPEs (regions of practical equivalence), we remain undecided for $\beta_{1,}$ since the 95% HDI of the posterior distribution is neither completely inside nor outside the ROPE. For β_2 we reject the null value (95% HDI of posterior distribution entirely outside ROPE).

> While the original analysis revealed an association of grip force amplitude with subjective value differences, this effect vanished when the grip response was described by a single measure (AUC). This shows that decomposition of the response trajectory via the gaussian model (and accounting for centroid and width) in fact increased sensitivity to detect associations with subjective value. Across all trials, AUC was positively correlated with the amplitude parameter of the Gaussian model (0.83), but only weakly correlated with the centroid and width parameters (0.25 and 0.45, respectively). Taken together, this suggests that modelling force trajectories using the gaussian model, and/or accounting for other aspects of the grip force response (width, centroid) increased sensitivity to detect associations with subjective value.

> Regressing AUC and the number of fixation shifts onto conflict based on the softmax choice probabilities (analogous to the analysis in section 3.4.1), provided no evidence for a relationship between AUC, fixation shifts and conflict (see Response Letter Figure 2). This is in agreement with the original analysis, in which we also found no evidence

that any of the regression coefficients for response vigour were greater than vs. smaller than zero (or vice versa).

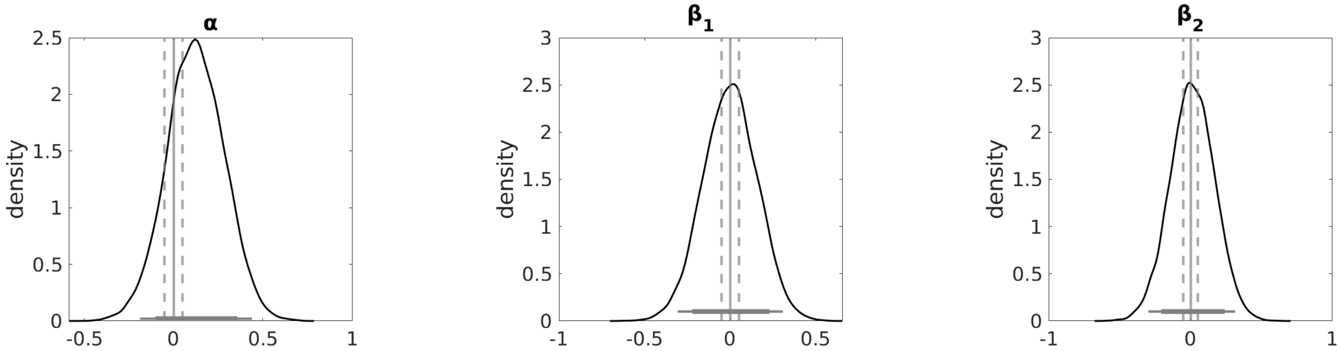

Figure 2. Hierarchical Bayesian regression results. Regression of area under the curve (AUC) and number of fixation shifts onto the trial-wise response conflict based on the choice probabilities (softmax model). Posterior distributions of the group-level parameter means. α: intercept, β_1: coefficient for amplitude, β_2: coefficient for fixation shift. Horizontal solid lines indicate the 85% and 95% highest density interval. Vertical solid lines indicate x = 0, and vertical dashed lines indicate the lower and upper bounds of the region of practical equivalence (ROPE).

5) The discussion is essentially a reiteration of the results, while it should provide perspectives for the conceptual advances and limitations for the interpretations.

> Thanks for suggesting to expand on further perspectives in the discussion. After summarising our findings in the first section of the discussion, we discuss the results against the background of our hypotheses. We discuss how our effects were produced (for instance that absolute value differences drive the observed effects) and place the results in the research context. We have revised the discussion in light of these suggestions, and expanded especially the section on dopamine and response vigour as well as the relevance and limitations sections (see section 4).

Minor concerns:

1) There are a lot of typos and mistakes throughout the manuscript, which should be checked carefully. For examples:
- Maximum conflict (page 9) should be 0.5 and not .05.
- Right graphs in Fig. 1 (page 12) should be labeled SMnorm and SNabs (and/or colored in grey and black)

- 'shift' should be deleted in the depiction of the DDMsig model in Table 2 (page 13)

> We thank the reviewer for the comments. We checked the manuscript for typing errors and corrected the data in Table 2 and 3 (section 3.3.1). We added the respective model names to the subtitles of the right graphs in Figure 1 and coloured the lines in black and grey, accordingly (section 3.2).

There are three parameters in the force response function but only two are reported in Table 7 and reported in Fig. 4 (page 19).

> Since we had no hypothesis with regard to effects on the width parameter of the grip response, we omitted the values. For the sake of completeness, we added the data to Table 7 and Figure 4 and 6.

Two studies are announced in the conclusion paragraph but three references are provided.

> We thank the reviewer for the hint and corrected the number of studies announced in the text (section 4):

"Three studies with human subjects also reported higher force production in states with augmented dopamine transmission [14, 15, 16]."

2) I would suggest to replace 'fixations' by 'visual fixation pattern' in the title, which would stand symmetrically to 'motor response vigor'. Also, these measures did not reflect 'subjective preferences', as suggested in the title, but value sum and difference (unsigned). This is critical because some passages seem to suggest that one can know which option is preferred by looking at grip force, while this is clearly not the case.

> In line with the reviewer's suggestion, we rephrased the title:

"Motor response vigour and visual fixation patterns reflect subjective valuation during intertemporal choice"

3) Doubling the number of participants required by the power calculation means that the power calculation was not taken seriously. I agree that for an explorative study, testing a new effect, power calculation is meaningless (because one cannot know the expected effect size). But then providing a power calculation sounds like showing off superficial rigor.

> The power analysis provides a lower bound for the sample size required for an experiment. We based our power analyses on previous studies reporting a magnitude effect for temporal discounting and handgrip force, respectively. This ensured sufficient power to replicate these effects. However, we did not have a specific a priori prediction regarding the effect size of a potential association between these measures. Therefore, we increased the sample size to have sufficient power to detect small effects.

4) The link with dopamine suggested in the discussion is interesting but not the only possibility. For instance, there is a literature relating conflict to noradrenalin (and anterior cingulate cortex) that the authors may want to cite.

> We thank the reviewer for the comment. Indeed, dopamine is not the only neurotransmitter involved in force production. We incorporated this into the discussion (section 4):

> “A number of studies suggest that the anterior cingulate cortex and its dopamin- ergic pathways are involved in the integration of effort and reward [50, 51, 52, 53]. […] In addition to dopamine, noradrenaline is also involved in force production [58] and conflict resolution [59, 60] . However, manipulating noradrenaline levels does not appear to affect reward sensitivity [61].”

5) The application to neuropsychiatric conditions is too far-fetched. Removing it would not undermine the interest of the study, in my opinion. Besides, that effort processing is impaired in Parkinson’s disease makes the grip force measure more problematic, not more relevant.

> Thanks for raising this issue. We consider the potential connection to neuropsychiatric conditions as highly relevant to the area being studied. As mentioned in our response to Reviewer #1 (see above) motivational disturbances are associated with a range of neuropsychiatric disorders. Therefore, revealing an implicit response vigour correlate of motivation in a task that has high clinical relevance (temporal discounting) can be informative in future work on psychiatric disorders. We agree that "disturbance" in effort processing is an important characteristic of Parkinson's disease, which constitutes a primary indication for a role of dopamine in this context. However, we respectfully do not agree with the conclusion that this renders the present grip force measure “more problematic, not more relevant”. After all, understanding alterations in a given process in a specific disorder such as Parkinson’s disease requires that the process of interest is first understood in the context of an intact neural system.

6) Some details are missing now and then.
- what is the unit for delays? (Presumably, days for the first and months for the last one)?

> The unit for delays is days. For the last delay item in the set, the last digit was missing (122 instead of 12). We adjusted the sentence accordingly (section 2.2):
>
> “[…] and six delay periods in days [1 7 13 31 58 122].”

- What is the force threshold that must be reached to confirm a choice?

> We added the information to the manuscript (section 2.3):
>
> “The force threshold to register a choice was set to 0.70 kgf. The threshold was determined in pilot measurements in such a way that false positive signals, caused by holding and slightly moving the force transducer, were avoided, while at the same time ensuring that no effort was required for a response.”

- How was conflict binned into 5 levels?

> We thank the reviewer for pointing out that we need to describe the analysis of response conflict in more detail. We have adjusted the section accordingly (section 2.5.2):

“For decision conflict based on the softmax model, we defined decision conflict from 1 (low conflict) to 5 (high conflict), with a probability of 0.5 of choosing the LL reward as maximum conflict. To provide a common scaling from low to high conflict, probabilities > 0.5 were ’flipped’ (1 - p), implying that for instance a probability of 0.1 and 0.9, respectively, of choosing the LL reward represent an equally low decision conflict (choose SS with high probability, and choose LL with high probability, respectively). The data were grouped into five bins using MATLAB’s discretize and accumarray function (choice probabilities between 0 and 0.1 assigned to bin 1, choice probabilities between 0.4 and 0.5 assigned to bin 5, etc.).”

Reviewer #3

In this paper Smith and Peters use an inter temporal choice task to examine how non-voluntary (or non-instrumental) vigor of a motor response can be influenced by subjective estimation of reward options. Several previous studies have shown links between reward processing and vigor of motor responses, with a possible mediator of Dopamine.
Vigor was here measured through grip-force, while eye tracking was used to examine visual fixation.

The two main findings were
1. There is a magnitude effect causing higher rewards to be discounted less, and with higher vigor
2. For easier trials (large subjective value differences between options) had larger vigor, shorter response times, and few shifts in fixation between the options.

Neither of these results are very surprising but the paper is well written, and the methods all seem very solid. The results are interesting, and worth publishing in PLoS Comp Bio.

It would have been interesting if more fine grained results could have been squeezed out of the eye tracking data, but that may be for future work (latency in saccade initiation? Pupilometry?).

> Pupillometry and the measurement of saccades were not the focus of the paper, but it would indeed be of interest to examine these measures in the context of the present task, and in relation to the grip force measures. However, our experimental setup, which required the participants to preselect an option by fixating it, likely restricted the overall gaze patterns. Therefore, we limited the analyses to the shifts of fixation. Further, since we did not specifically construct isoluminant stimuli, the analysis of pupil dilation would be confounded by differences in luminance between the stimuli and conditions. We added these point to the limitations section (section 4), where we now write:
>
> "For the present task, it would have been interesting to also include pupillometry and more comprehensive analyses of e.g. saccade reaction times and velocities [71, 19]. Since the usage of a force transducer functions as a single key, some method of preselecting one of two options was necessary. Choice selection was thus implemented such that an option was preselected by visual fixation and selected by subsequently pressing the force transducer. Hence, an option could only be chosen if it was concurrently fixated, which may have restricted the fixation patterns. Therefore, we limited the analyses to the shifts of fixation. Further, since we did not specifically construct isoluminant stimuli, the analysis of pupil dilation would be confounded by differences in luminance between the stimuli and conditions."

I don’t have any major comments, but just wanted to raise on potential issue with the model specification.
Twelve parameters (for the largest model) is a lot, making me a bit worried about over-fitting. But as DIC is meant to correct for that, I will take the numbers at face value.

> Thanks for raising this issue. While models with a smaller DIC are preferred over models with larger DIC values, DIC also penalises a model’s fit by its complexity/number of parameters (Spiegelhalter et al., 2014, J R Stat Soc Series B Stat Methodol). If the superior model fit of the non-linear DDM mainly reflects overfitting, this should manifest itself in poor parameter recovery. However, in previous work, we looked at parameter recovery of the very same drift diffusion model, including shift parameters for the effects of magnitude, showing that for both the “baseline” and the shift parameters, the group-level parameters recovered well (Wagner et al., 2020, J Neurosci).

I have some minor comments:
Page 6
“ sixteen percentages of the SS reward value “ are these percentages or ratios?

> We thank the reviewer for noticing the mix-up. The values given are ratios of the reward values. We adjusted the sentence accordingly:
>
> “[…] the LL reward consisted of combinations of sixteen ratios of the SS reward value [1.03 1.05 1.10 1.15 1.20 1.25 1.35 1.45 1.50 1.70 1.90 2.20 2.50 2.90 3.30 3.80]”

Are the delay periods really [1 7 13 31 58 12], i.e. 12 as the last? Are these given in seconds? Hours? Days?

> The unit for delays is days. For the last delay item in the set, the last digit was missing (122 instead of 12). We corrected the sentence accordingly (section 2.2):
>
> “[…] and six delay periods in days [1 7 13 31 58 122].”

“track the gaze patterns and to give real-time feedback to the participant,” what feedback was given? Or was it just to highlight the current fixated reward option

> The feedback was to highlight the currently fixated option. We rephrased the sentence to make this more clear:
>
> “For this purpose, we used an eyetracking system (SensoMotoric Instruments, Model: RED 500, sampling rate: 500 Hz) to track the fixation patterns and highlight the currently fixated reward option in real-time.“

“All logfiles were checked for stereotypic response patterns” should that be non-stereotypic? Maybe rephrase

> The term stereotypic may be misleading in this context. We rephrased the paragraph and provided more information on the subject matter:

“All logfiles were checked for stereotypic response patterns (exclusively SS or LL choices), none were found. Choice patterns consisting of exclusively SS or LL choices may indicate that the participants proceeded heuristically rather than including values and delay periods in their reasoning.”

Incidentally, having a figure showing the experimental setup (even if very simple) could be useful

> Since the setup solely consisted of a computer and screen, an eyetracking device mounted below the screen, and a force transducer, we feel that an additional figure would not be very informative. We agree that the manuscript would benefit from a more comprehensive description of the experimental setup and therefore we split the task and data collection sections into task and experimental setup and described the experimental setup in greater detail (section 2.3):

“The measurements took place at the Psychology Department of the University of Cologne. During testing, the participants were seated in a dimly lit, electrically and acoustically shielded room, with their head placed in a chinrest. Prior to the experiment, they were instructed to press the handgrip with maximal force three times in succession with their dominant hand. The procedure was disguised as calibration procedure. Following that, the participants were instructed that the level of force exerted is irrelevant to the task structure.

After presenting both options, one of the two options could be preselected through visual fixation. For this purpose, we used an eyetracking system (SensoMotoric Instruments, Model: RED 500, sampling rate: 500 Hz) to track the fixation patterns and highlight the currently fixated reward option in real-time. For highlighting the fixated option, the corresponding screen areas were defined as follows: Left area <= 1 10 screen pixels of x-coordinate x 4, middle area >= 1 10 screen pixels of x-coordinate x 4 and <= 1 10 screen pixels of x-coordinate x 6, right area >= 1 10 screen pixels of x-coordinate x 6.

The responses were logged using a hand dynamometer measuring grip force (BIOPAC Systems, Inc., Model: TSD121C, isometric range: 0 - 100 kgf). The force threshold to register a choice was set to 0.70 kgf. The threshold was determined in pilot measurements in such a way that false positive signals, caused by holding and slightly moving the force transducer, were avoided, while at the same time ensuring that no effort was required for a response. There was no response time limit. After having pre-selected an option through visual fixation, participants could still deliberate and decide for the other option as long as the force transducer had not been pressed. The measured variables included the participants’ choices, response times, fixation shift patterns and handgrip force applied during response selection, as well as their maximum handgrip force.”

Page 9

I did not understand how the decision conflict was operationalised as a number from 1 to 5. Please explain

> We thank the reviewer for pointing out that we need to describe the analysis of response conflict in more detail. We thus provided further information on the procedure (section 2.4.2).:
>
> "For decision conflict based on the softmax model, we defined decision conflict from 1 (low conflict) to 5 (high conflict), with a probability of 0.5 of choosing the LL reward as maximum conflict. To provide a common scaling from low to high conflict, probabilities > 0.5 were 'flipped' (1 - p), implying that for instance a probability of 0.1 and 0.9, respectively, of choosing the LL reward represent an equally low decision conflict (choose SS with high probability, and choose LL with high probability, respectively). The data were grouped into five bins using MATLAB's discretize and accumarray function (choice probabilities between 0 and 0.1 assigned to bin 1, choice probabilities between 0.4 and 0.5 assigned to bin 5, etc.)."

Page 11

The variable d is used for both the grip model and the number of fixation shifts, please change one of them.

> We thank the reviewer for noticing the duplicate use of the variable name and renamed the variable "d" (offset) of the grip response model to "h" (section 2.5.3).

Page 12

Figure 1, make clearer that the two right most plots are for the same parameter but different models.

> We thank the reviewer for the suggested improvement. We added the respective model names to the subtitles of the right graphs in Figure 1 and have colour-coded the lines in black and grey, accordingly (section 3.2).

Page 15

Figure 2, I would suggest to make this bigger

Also, are the RTs split by L/R? I.e. why are there negative RTs?

> To render the figure more readable, we increased the height of the plot and each subplot. (Figure 2, section 3.3.2 and Figure S1, supplementary material). The negative RTs arise from the boundary definitions of the DDM. We defined the lower boundary as choosing the SS reward, and the upper boundary as choosing the LL reward. For this purpose, choices towards the lower boundary were multiplied by -1. The negative RTs

in Figure 2 thus indicate SS choices. To make this more clear, we added these details to the figure caption (Figure 2, section 3.3.2 and Figure S1, supplementary material).

Page 18

Table 6 and Fig 3, why report the centroid in number of samples instead of time?

> We changed the reporting of the centroid and width from number of samples to seconds and adjusted all respective tables and figures accordingly (Table 6 and 7, Figure 3, 4, S2 and S3).

Also, is there a reason gap force width is not reported here?

> Since we had no hypothesis with regard to effects on the width parameter of the grip response, we omitted the values. For the sake of completeness, we added the data to Table 7 and Figure 4 and 6.

For the discussion on Dopamine or future work: The authors may also find Beierholm et al 2013 (https://www.nature.com/articles/npp201348) interesting, showing that Dopamine modulated reward-related vigour in a reaction time task, thus building on the work by Niv et al 2007 (https://link.springer.com/article/10.1007/s00213-006-0502-4).

> We thank the reviewer for providing the reference and incorporated it into the discussion (section 4):

“ In a rewarded odd-ball discrimination task, Beierholm and colleagues [55] demonstrated that L-DOPA modulated reward-related response vigour (reaction times). The results suggest that the influence of reward rate on response vigour is mediated by dopamine transmission.”

A few of the references are incomplete, e.g. Moreira & Barbosa, as well as Schultz

> We thank the reviewer for the hint and checked our references for missing information.

Reviewer #4

This study investigates whether response vigor serves as an additional implicit measure of subjective utility during intertemporal choice. They find that motor response vigor reflects the difference in subjective value between the options and is also includence by the absolute value of the options. Subject grasp earlier and with more force the greater the difference int subjective value between the options and to some extent, the greater the absolute value of the two options.

This is an interesting and novel study. It has been previously shown that relative saccade vigor during deliberation between two options correlates with the difference in subjective value between the options. This study shows that grasp vigor when selecting between two options also correlates with the difference in subjective value. This demonstrates that vigor of multiple effectors (eyes, hand) may simultaneously reflect subjective utility. Overall, I am excited to see these results. However, I have a few comments and suggestions that I believe would strengthen and extend their findings.

General Comments

1. I am not sure why the authors do not simply measure peak grasp force as their main vigor measure. Instead there is a model that is fit to the profiles. I understand that they show there is a good fit between model and data, but It would be helpful if they also performed their same analysis using peak grasp force.

> Thanks for raising this issue – a similar point was also raised by Reviewer #2, who suggested to look at area under the curve (AUC) of the force trajectory. Following these suggestions, we conducted additional analyses using two model-free measures for the grip response, AUC and peak force. As in our main analysis, we regressed grip force and the number of fixation shifts onto the subjective value differences, replacing the parameters of the Gaussian grip force model by AUC and peak force (maximum force per trial), respectively.

> In short, replacing the 3-parameter gaussian model with AUC yielded no effects, suggesting that the gaussian model is more sensitive, and/or accounting for other aspects of the grip force trajectory (width and centroid) increases the sensitivity to detect associations with subjective value. Regressing peak force and the number of fixation shifts onto the subjective value differences revealed similar results to our main analysis.

(1) AUC

> As noted in our response to Reviewer #2 above, we computed the area under the curve (AUC) for each trial via the approximate integral of the measured trial-wise force trajectory by means of trapezoidal integration using MATLAB's trapz function. We then regressed AUC and the number of fixation shifts onto the subjective value differences (that is, we replaced the parameters of the Gaussian grip force model by AUC). The results can be seen in Response Letter Figure 3.

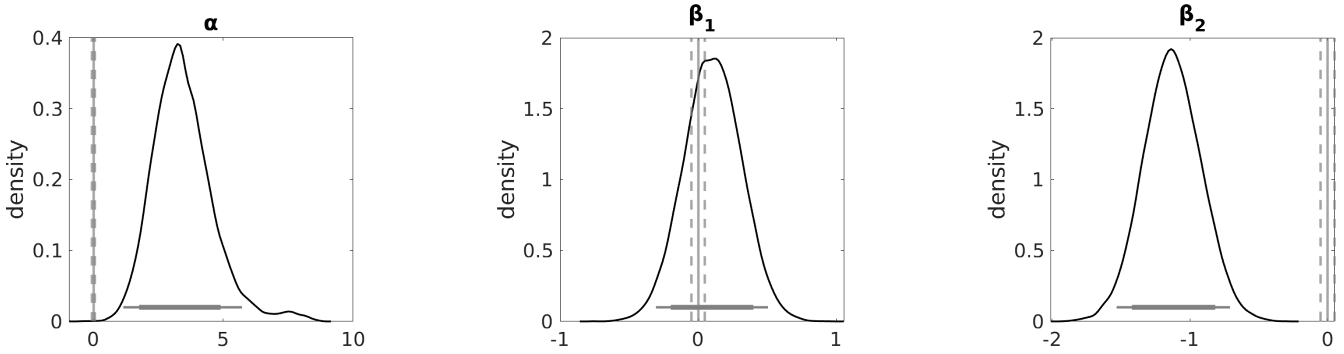

Figure 3. Hierarchical Bayesian regression results. Regression of area under the curve (AUC) and number of fixation shifts onto the value differences (DDM). Posterior distributions of the group-level parameter means. α: intercept, β_1: coefficient for AUC, β_2: coefficient for fixation shift. Horizontal solid lines indicate the 85% and 95% highest density interval. Vertical solid lines indicate x = 0, and vertical dashed lines indicate the lower and upper bounds of the region of practical equivalence (ROPE).

> The Bayes factor for the regression coefficient for AUC provides only anecdotal evidence for values greater than zero vs. smaller than zero (BF for β_1: 2.27). The Bayes factor for the regression coefficient for the number of fixation shifts provides extreme evidence for values smaller than zero vs. greater than zero (BF for β_2: > 10^{308}). Looking at the ROPEs (regions of practical equivalence), we remain undecided for $\beta_{1,}$ since the 95% HDI of the posterior distribution is neither completely inside nor outside the ROPE. For β_2 we reject the null value (95% HDI of posterior distribution entirely outside ROPE).

> While the original analysis revealed an association of grip force amplitude with subjective value differences, this effect vanished when the grip response was described by a single measure (AUC). This shows that decomposition of the response trajectory via the gaussian model (and accounting for centroid and width) in fact increased sensitivity to detect associations with subjective value. Across all trials, AUC was positively correlated with the amplitude parameter of the Gaussian model (0.83), but only weakly correlated with the centroid and width parameters (0.25 and 0.45, respectively). Taken together, this suggests that modelling force trajectories using the gaussian model, and/or accounting for other aspects of the grip force response (width, centroid) increased sensitivity to detect associations with subjective value.

> Regressing AUC and the number of fixation shifts onto conflict based on the softmax choice probabilities (analogous to the analysis in section 3.4.1), provided no evidence for a relationship between AUC, fixation shifts and conflict (see Response Letter Figure 4). This is in agreement with the original analysis, in which we also found no evidence that any of the regression coefficients for response vigour were greater than vs. smaller than zero (or vice versa).

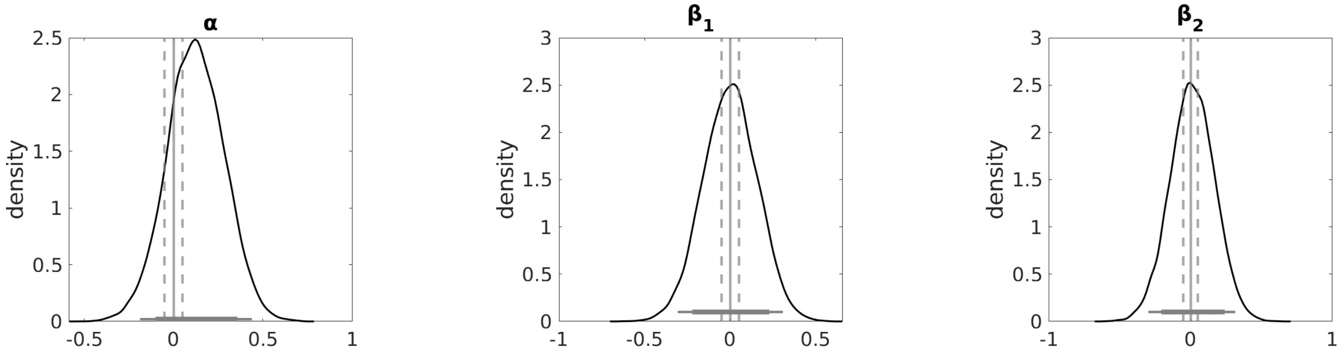

Figure 4. Hierarchical Bayesian regression results. Regression of area under the curve (AUC) and number of fixation shifts onto the trial-wise response conflict based on the choice probabilities (softmax model). Posterior distributions of the group-level parameter means. α: intercept, β_1: coefficient for amplitude, β_2: coefficient for fixation shift. Horizontal solid lines indicate the 85% and 95% highest density interval. Vertical solid lines indicate x = 0, and vertical dashed lines indicate the lower and upper bounds of the region of practical equivalence (ROPE).

(2) Peak force

> In contrast to AUC, which showed a correlation of .83 with the model-based amplitude, peak force showed a correlation of .99 with the model-based amplitude. Regressing peak force and the number of fixation shifts onto the subjective value differences (see Response Letter Figure 5) revealed similar results to our main analysis (section 3.4.1).

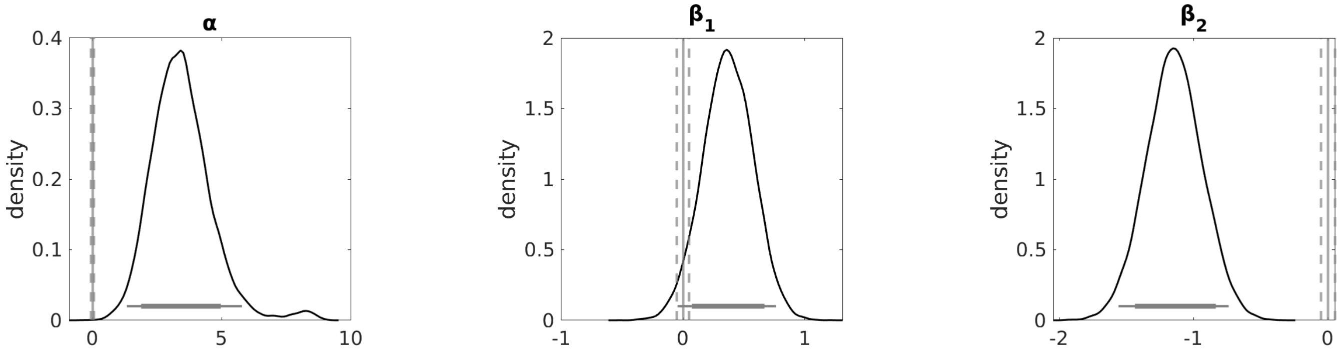

Figure 5. Hierarchical Bayesian regression results. Regression of peak force and number of fixation shifts onto the value differences (DDM). Posterior distributions of the group-level parameter means. α: intercept, β_1: coefficient for peak force, β_2: coefficient for fixation shift. Horizontal solid lines indicate the 85% and 95% highest density interval. Vertical solid lines indicate x = 0, and vertical dashed lines indicate the lower and upper bounds of the region of practical equivalence (ROPE).

> The Bayes factor for the regression coefficient for peak force provides strong evidence for values greater than zero vs. smaller than zero (BF for β_1: 26.07), recapitulating our original results. The Bayes factor for the regression coefficient for the number of

fixation shifts provides extreme evidence for values smaller than zero vs. greater than zero (BF for β_2: $> 10^{308}$). Looking at the ROPEs (regions of practical equivalence), we remain undecided for β_1, since the 95% HDI of the posterior distribution is neither completely inside nor outside the ROPE. For β_2 we reject the null value (95% HDI of posterior distribution entirely outside ROPE).

> Looking at the correlations between AUC and peak force and the parameters of the Gaussian grip force model, both AUC and peak force are highly correlated with the amplitude parameter of the Gaussian model (0.83 and 0.99, respectively), but only weakly correlated with the centroid and width parameter (0.25 and 0.45, respectively). Taken together with the analysis of the AUC, this shows that peak force (being highly correlated with amplitude) reproduces the amplitude effects better that AUC. Furthermore, additionally controlling for other aspects of the force trajectory (width, centroid) yielded further improvements in sensitivity to detect associations with subjective value. Since the analyses did not provide further insights, we present them in the response letter only.

a. The authors could also consider looking at rate of grasp force development and time to required force as additional measures of vigor.

> Regarding rate of grasp: this is a good point. However, the rate of grasp force development is directly proportional to the width of the gaussian, which was accounted for and included in our models.

> Time to required force is basically the response time, which essentially corresponds to the modelled centroid parameter of the grip force model. Indeed, the correlation between the response times as registered at the stimulus presentation computer and the centroid parameter estimated using the Gaussian grip force model was 0.996, showing a very close correspondence. In the light of such a high correlation, we believe that a separate analysis of time to required force is unlikely to yield additional insights.

2. I think the magnitude effect on response vigor is quite interesting. As I understand it the magnitude effect suggests that for the two pairs of options with the same relative difference in subjective value, the pair with the greater magnitude will have a greater response vigor. Related to this, I have the following questions:
a. The authors seems to suggest that the magnitude effect on response vigor is in addition to the effect of the difference in subjective value, which is potentially very interesting. However, could the increase in vigor in the high magnitude condition be due to greater relative values differences between options in high magnitude condition compared to the low magnitude condition?

> Thanks for giving us the opportunity to clarify this. The relative value differences were the same for both conditions, since the LL rewards were multiples of the SS rewards. However, the associations between value differences and response vigour were only observed in the model using absolute (not normalised) value differences. This suggests that this effect is driven by trials with large absolute value differences, rather than relative value differences.

b. If indeed there is a magnitude effect then this could potentially be seen across all trials and not just between conditions. Do the authors see an effect of the total value (sum of both options) on response vigor?

> This is an important point. Following the reviewer's suggestion, we analysed the association between response vigour and total value. To this end, we regressed grip force and the number of fixation shifts onto the sum of the LL and SS option amounts (model 1) and onto the sum of the subjective LL and SS option values (model 2), based on the discount rates estimated from the drift diffusion model.

Model 1: Sum of LL and SS amounts (model-free)

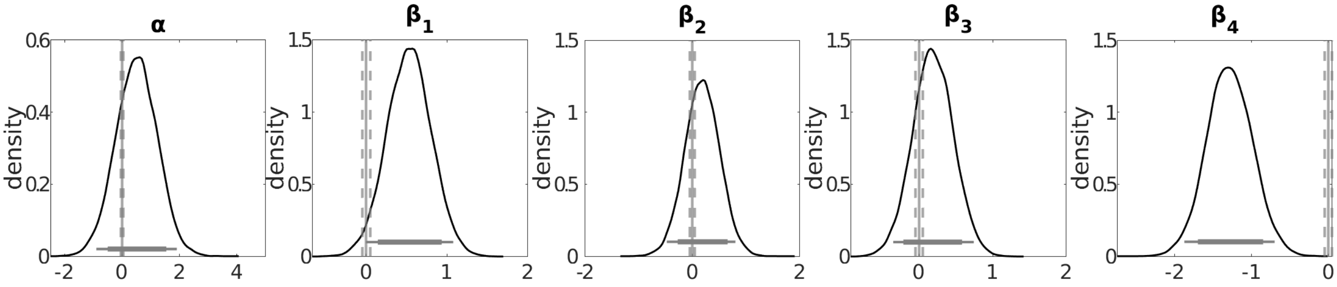

Figure 5. Hierarchical Bayesian regression results. Regression of the parameters of the Gaussian-modelled grip force response and number of fixation shifts onto the total sum of the option amounts (model-free). Posterior distributions of the group-level parameter means. α: intercept, β_1: coefficient for grip force amplitude, β_2: coefficient for grip force centroid, β_3: coefficient for grip force width, β_4: coefficient for fixation shift. Horizontal solid lines indicate the 85% and 95% highest density interval. Vertical solid lines indicate x = 0, and vertical dashed lines indicate the lower and upper bounds of the region of practical equivalence (ROPE).

> The medians of the group-level posterior distributions were as follows: α = 0.51 (intercept), β_1 = 0.53 (amplitude), β_2 = 0.19 (centroid), β_3 = 0.19 (width), β_4 = -1.29 (N gaze shifts) (see Response Letter Figure 5). The Bayes factor for the regression coefficient for peak force provides very strong evidence for values greater than zero vs. smaller than zero (BF for β_1: 41.07). The Bayes factors for the coefficients for centroid and width provide only anecdotal and moderate evidence, respectively, for values greater than zero vs. smaller than zero (BF for β_2: 2.79, BF for β_3: 3.04). The Bayes factor for the coefficient for number of fixation shifts provides extreme evidence for values smaller vs. greater than zero (BF for β_4: > 10^{308}). For β_1, β_2 and β_3 we remain undecided, since the 95% HDI of the posterior distribution is neither completely inside

nor outside the ROPE. For β_4 we reject the null value (95% HDI of posterior distribution entirely outside ROPE).

Model 2: Sum of subjective option values (based on DDM)

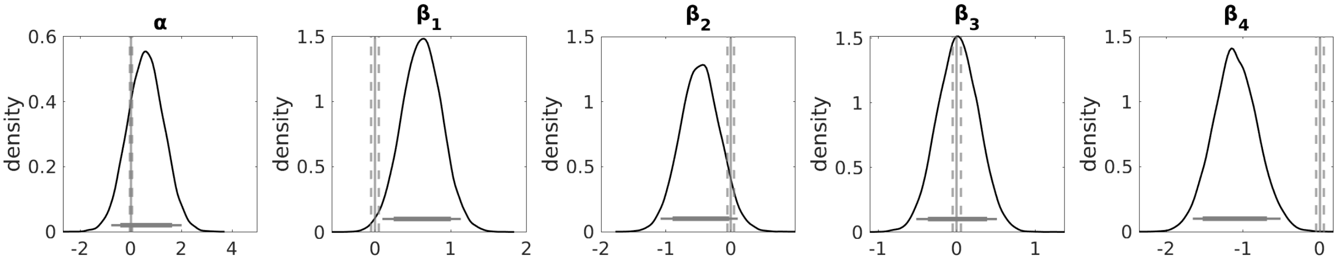

Figure 6. Hierarchical Bayesian regression results. Regression of the parameters of the Gaussian-modelled grip force response and number of fixation shifts onto the total sum of the subjective option values from the drift diffusion model (DDM). Posterior distributions of the group-level parameter means. α: intercept, β_1: coefficient for grip force amplitude, β_2: coefficient for grip force centroid, β_3: coefficient for grip force width, β_4: coefficient for fixation shift. Horizontal solid lines indicate the 85% and 95% highest density interval. Vertical solid lines indicate x = 0, and vertical dashed lines indicate the lower and upper bounds of the region of practical equivalence (ROPE).

> The medians of the group-level posterior distributions were as follows: α = 0.59 (intercept) β1 = 0.62 (amplitude), β2 = -0.47 (centroid), β3 = 0.01 (width), β4 = -1.11 (N gaze shifts) (see Response Letter Figure 6). The Bayes factor for the regression coefficient for amplitude provides extreme evidence for values greater than zero vs. smaller than zero (BF for β_1: 111.18). For the centroid coefficient, the Bayes factor provides strong evidence for values smaller than zero vs. larger than zero (BF for β_2: 16.37). For the width coefficient, the Bayes factor provides only anecdotal evidence for values greater than zero vs. smaller than zero (BF for β_3: 1.04). The regression coefficient for fixation shifts provides extreme evidence for values smaller than zero vs. larger than zero (BF for β_4: 10933.53).

> Generally, these analyses confirm what this Reviewer suspected. The effects indeed can be reproduced when focusing on total value. In line with the idea that subjective (rather than objective) option dimensions shape behaviour, the associations with amplitude and centroid were more pronounced for model 2 that used subjective (DDM-based) values for the computation of total value. We now included the results from model 1 in the supplement (section S3), and included model 2 in the main paper (section 3.4.2), as we believe it provides helpful converging evidence that the results are robust against specifics of the analysis, and indeed that total value effects might even show the closest association with grip force amplitude. Of course we point out that this specific model was not preregistered.

Minor Comments
1. Please provide additional detail about the deliberation phase of the experiment. For example, how much time did the subjects have to indicate their choice?, how much time was allowed to pass between pre-selection and the grasp response?

> We provided further information on the deliberation phase (section 2.3):

“There was no response time limit. After having pre-selected an option through visual fixation, participants could still deliberate and decide for the other option as long as the force transducer had not been pressed.”
