# Supplementary material for: Motor response vigour and visual fixation patterns reflect subjective valuation during intertemporal choice
Source: PLoS Comput Biol. 2022 Jun 10;18(6):e1010096. doi: 10.1371/journal.pcbi.1010096 (PMC9187114; doi:10.1371/journal.pcbi.1010096)
Supplement: Attachment — Submitted filename: Response_to_reviewers.pdf [file pcbi.1010096.s005.pdf]

Reviewer #1: The authors have provided thoughtful responses to all of the questions I have previously raised. I also believe that the authors have done a great job in revising the manuscript to include additional explanation of their analytical frameworks, results, and discussion. The present manuscript addresses an important and timely topic which will have a wide-reaching impact in field of decision-making and human cognition.

I only have one further comment which I hope the authors could help clarify – in your response about t0. I'm not sure I necessarily agree with this statement: "In particular, if all trials were included, the non-decision time would necessarily adjust, shifting the modeled RT distribution towards zero as much as required to ensure a probability density > 0 for fast outlier trials." Could the author please expand on this idea a bit more? Also, relatedly, I'm wondering if the authors think t0 could inform us something meaningful across experiments that use the same exact stimulus setup but with different response schemes (e.g., gaze shifting/grip force vs button press vs continuous dial response)?

> We thank the reviewer for pointing out that this needs to be described in greater detail. The reason is that even implausibly fast outlier trials (e.g. 50 ms) must be assigned a probability density > 0, leading to a corresponding adjustment in particular of the non-decision time t0. Put simply, the modelled RT distribution for a given participant is shifted towards 0 as much as required to accommodate even the fastest observed RT. In our experience, this can lead to poor model fits at the level of individual participants, and consequently also impacts on the fit of hierarchical models. We therefore excluded physiologically implausible response times below 200 ms, to ensure that such responses do not exert an undue influence on model parameters and fit. We discuss this in more detail in section 2.4.1 (Preprocessing):

> "Valid response times are physiologically limited to a lower bound of around 100 to 200 ms [32, 33 ]. Since even implausibly fast outlier trials must be assigned a probability density > 0, modelled response time distributions for a given participant are shifted towards zero as much as required to accommodate for such response times. This may lead to poor model fits at the level of individual participants, and consequently may also impact on the fits of hierarchical models. Therefore, we excluded trials with response times below 200 ms."

With regard to the second point, provided that the data are preprocessed and analysed in the same way, comparing t0 across experiments that use different response schemes or modalities may yield information regarding effects of response schemes on stimulus encoding, preparation and execution of the motor response. This is exactly what we recently observed in a study in which decision tasks were performed in VR and responses logged via VR controllers (Bruder et al., Scientific Reports, 2021). However, which of these processes is affected cannot be inferred from t0 alone, since t0 reflects both motor and non-motor aspects. However, there is preliminary work on

the decomposition of the non-decision time of drift diffusion models using electro-myographical activity (Weindel et al., 2021, PsyArXiv). From the results, the authors conclude that stimulus encoding does not necessarily end when evidence accumulation begins, and that the onset of the motor response does not necessarily denote the end of the deliberation process. Further, the authors assume that t0 must be interpreted differently depending on the participants' adjustments in response to task instructions. Therefore, a meaningful comparison of non-decision times may only be made if all experimental parameters except e.g. the response scheme (as suggested above) are kept constant. We expand on this in the Discussion (section 4):

> "In general, the estimated non-decision times τ were longer than in typical laboratory experimental setups (> 1000 ms) [21, 31]. The non-decision time parameter τ models time that is not related to the decision process, such as stimulus encoding and motor response execution. Our estimated non-decision times were comparable to the estimated non-decision times from a recent study using a VR environment, where the participants logged their responses using VR-compatible controllers, as opposed to simple response keys [47]. This is likely due to the task's requirement of first preselecting an option through visual fixation before finally selecting it using the hand dynamometer. However, since τ reflects both motor and non-motor components, which of these processes is affected cannot be inferred from τ alone. There is preliminary work on the decomposition of the non-decision time of drift diffusion models using electro-myographical activity [48]. The authors conclude that stimulus encoding does not necessarily end when evidence accumulation begins, and that the onset of the motor response does not necessarily denote the end of the deliberation process. Further, the non-decision time may be influenced by participants' adjustments in response to task instructions. Therefore, a meaningful comparison of non-decision times across experiments with different response schemes may only be made if all other experimental parameters are kept constant."

Reviewer #2: The authors did a great job in their revision. I still think the paper could be clearer at the conceptual level, but the analyses are technically sound and the results certainly deserve publication. There is no need to prolong this review process with more iterations, but here is a couple of points that could be improved:

1) The paper is introduced with the notion of effort discounting (how a reward is devalued by the effort required to get it), which is not the question of interest in this paper. The authors justify this introduction by stating that their paper reveals another aspect of effort – in addition to reflecting a cost, it would reflect motivational process. But the two aspects are the two sides of the same coin: if motivation is measured by the cost one is willing to pay for a given reward, then accepting a higher cost (exerting more effort) reveals stronger motivation without changing the basic notion that effort is a cost. In other words, the literature on effort

discounting also uses effort to reveal motivation, there is no novelty here. The difference, as stated later by the authors, is that effort is an implicit measure in the present study because it is not instrumental in getting a reward. The question of interest (whether implicit measures can provide further insight into subjective valuation during decision-making) without the misleading detour through effort discounting.

> Following the reviewer's comment, we have removed all notions of effort discounting from the text (see Introduction and Discussion, sections 1 and 4).

2) The interest of the approach is sold with the perspective of applications to neuropsychiatric diseases. I know everyone does that and I'm not particularly shocked, except by the justification that response vigor (grip force) measurement would be informative in conditions where effort processing is impaired (like Parkinson's disease). To put it bluntly, this is like saying that eye-tracking would be helpful to investigate blind people because their visual exploration is impaired. The paper is interesting enough to attract attention without using this sort of twist.

> Following the reviewer's suggestion, we removed the notion that response vigour may be informative in patient populations with impaired effort processing/motivational deficits (see Author Summary and section 4, Discussion).

Reviewer #3: I am happy with the changes

Reviewer #4: Thank you to the authors for their responses and additional analysis. I have a few additional comments:

1) Please report the times (mean, sd) observed between preselection and choice registration with the force transducer. This is useful information for future studies investigating similar questions. For example, one could ask how close does the time of the decision need to be to the motor response for this vigor effect to be observed. If the motor response is much delayed (say 1 min) would it still reflect the decision variables? Thus providing the numbers observed in this study will be helpful for others.

> Thanks, this raises an interesting issue. We analysed the times between preselection and choice, i.e. the period between the choice and the last fixation of the selected option. On average, the time was 0.03 seconds (*SD* = 0.11, range = 0.001 to 3.383). In summary, the time between preselection and choice was smaller than 0.5 seconds in 98.83% of trials, and greater than 1 second in 0.24% of trials. Thus, it can be said that in most cases the motor response occurred very shortly after the last fixation. We added the information to the main text (see Results, section 3.4).
>
> > "The time between visual preselection of an option and choice registration with the grip force transducer was on average 0.03 seconds (*SD* = 0.11)."

2) I am not clear what the second hypothesis is stating. As written, it says: "(ii) Subjective utility modulates response times and grip force: Faster response times and stronger effort (handgrip force) for choices with high utility (subjective value)." It seems they mean 'the difference in subjective utility' rather than simply 'subjective utility'? If not, then please clarify subjective utility of which option will modulate response times and grip force.

> We thank the reviewer for pointing out that the hypothesis needs to be formulated more precisely. We rephrased the hypothesis as follows (see section 1, Introduction):

> "Differences in subjective utility modulate response times and grip force: Faster response times and stronger effort (handgrip force) in trials with high subjective value differences."

3) In section '3.4.2 Value sum effect', please add a sentence at the end of the paragraph to summarize the main findings.

> In line with the reviewer's suggestion, we summarise the results at the end of section 3.4.2:

> "Accordingly, this analysis shows higher grip force amplitudes, faster response times and fewer fixation shifts for trials with high value sums across the options."

4) The results are a little difficult to follow. It would be helpful if the authors let the readers know when results supported their hypotheses (and specificy which hypothesis). Even better, would be a summary at the end of the results or in the discussion where there was a recap of the hypotheses and what was supported or rejected.

> We indicate whether a hypothesis (and which) was confirmed or not and added a paragraph to the Discussion (section 4), providing a recap of the findings with respect to our hypotheses:

> "Summarising the findings with respect to our hypotheses, as expected, participants discounted rewards as a function of delay (hypothesis i). In accordance with our predictions, we further found evidence that differences in subjective utility modulated response times and grip forces, such that response times decreased and grip forces increased with increasing subjective value differences between options (hypothesis ii). Unlike what we thought, we found no relationship between decision conflict and grip force, response time or fixation shifts (hypothesis iii). Yet, in line with our hypothesis, higher rewards were discounted less and elicited stronger effort (magnitude effect). Contrary to our hypothesis, however, the response times were not significantly different between the two magnitude conditions (hypothesis iv)."

5) Can the authors clarify whether the new analysis of the effect of total value on response vigor hold true even within a condition? Is the effect they observe driven by the condition

effect (high vs low), or is this something observed even with the low condition or high condition, independently?

> To address this question, we performed separate regression analyses for each magnitude condition (see below). In summary, the effects of centroid and number of fixation shifts are found in both regression models, but are more pronounced in the model including the high-magnitude condition only. The amplitude effect is only found in the regression model based on the high-magnitude condition. The results are consistent with our previously described assumption that the observed associations between subjective values, grip force parameters and fixation patterns are driven by trials with large absolute value differences or in this case, sums, respectively, and further mirror the magnitude effect. We added the analyses to the Supplement (section S4) and refer to them in the main text (see Results, section 3.4.2):
>
> "Running separate regressions on value sums of the high and low magnitude condition revealed that this effect was driven by the high magnitude condition (see section S4 of the supplementary material.)"

Model 1: Sum of subjective option values (based on DDM) for the low magnitude condition

> The medians of the group-level posterior distributions were as follows: $\alpha$ = 0.94 (intercept), $\beta1$ = 0.12 (amplitude), $\beta2$ = -0.30 (centroid), $\beta3$ = -0.19 (width), $\beta4$ = -0.56 (N fixation shifts) (see Response Letter Figure 1). The Bayes factor for the regression coefficient for grip force amplitude provides anecdotal evidence for values greater than zero vs. smaller than zero (BF for $\beta_1$: 2.81). The Bayes factor for the regression coefficient for the grip centroid provides strong evidence for values smaller than zero vs. greater than zero (BF for $\beta_2$: 10.40), the Bayes factor for the regression coefficient for the width parameter provides moderate evidence for values smaller than zero vs. greater than zero (BF for $\beta_3$: 4.73), and the Bayes factor for the regression coefficient for the number of fixation shifts provides extreme evidence for values smaller than zero vs. greater than zero (BF for $\beta_4$: 184.48). Since the 95% HDIs of the posterior distributions for $\beta_1$, $\beta_2$, and $\beta_3$ fall neither completely inside nor outside the ROPE, we remain undecided for these three regression coefficients. For $\beta_4$ we reject the null value (95% HDI of posterior distribution entirely outside ROPE).

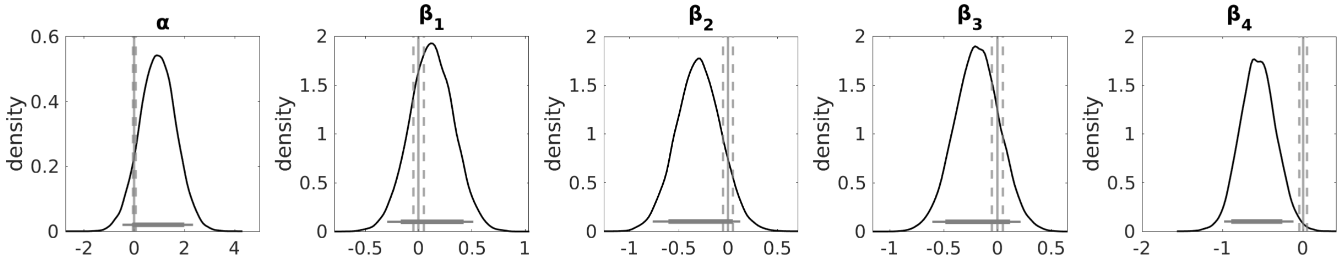


Figure 1. Hierarchical Bayesian regression results. Regression of the parameters of the Gaussian-modelled grip force response and number of fixation shifts onto the total sum of the subjective option values from the drift diffusion model (DDM) for the low magnitude condition. Posterior distributions of the group-level parameter means. α: intercept, $\beta_1$: coefficient for grip force amplitude, $\beta_2$: coefficient for grip force centroid, $\beta_3$: coefficient for grip force width, $\beta_4$: coefficient for fixation shift. Horizontal solid lines indicate the 85% and 95% highest density interval. Vertical solid lines indicate x = 0, and vertical dashed lines indicate the lower and upper bounds of the region of practical equivalence (ROPE).

Model 2: Sum of subjective option values (based on DDM) for the high magnitude condition

> The medians of the group-level posterior distributions were as follows: α = 0.99 (intercept), β1 = 0.53 (amplitude), β2 = -1.18 (centroid), β3 = 0.11 (width), β4 = -0.83 (N fixation shifts) (see Response Letter Figure 2). The Bayes factor for the regression coefficient for grip force amplitude provides very strong evidence for values greater than zero vs. smaller than zero (BF for $\beta_1$: 60.27). The Bayes factor for the regression coefficient for the grip centroid provides extreme evidence for values smaller than zero vs. greater than zero (BF for $\beta_2$: 2181.35), the Bayes factor for the regression coefficient for the width parameter provides moderate evidence for values greater than zero vs. smaller than zero (BF for $\beta_3$: 2.19), and the Bayes factor for the regression coefficient for the number of fixation shifts provides extreme evidence for values smaller than zero vs. greater than zero (BF for $\beta_4$: 593.96). For $\beta_2$ and $\beta_4$ we reject the null value (95% HDI of posterior distribution entirely outside ROPE). For $\beta_1$ we also reject the null value, since the 95% HDI dos not include zero and only 0.63% of the 95% HDI overlaps with the ROPE. For $\beta_3$ we remain undecided, since the 95% HDI of the posterior distribution is neither completely inside nor outside the ROPE.

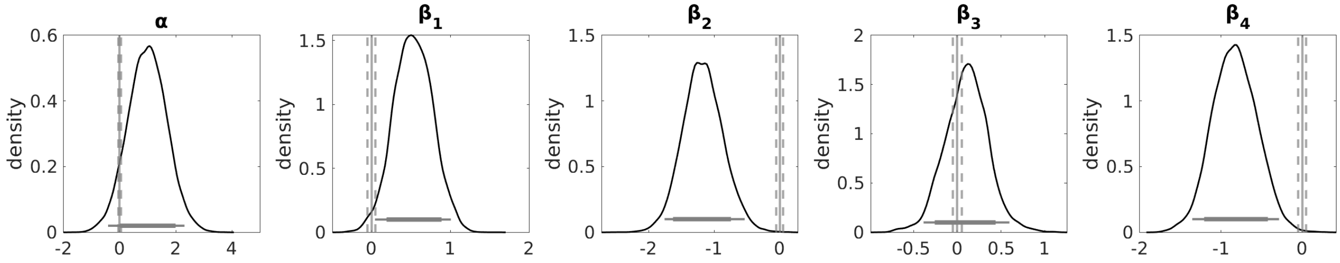


Figure 2. Hierarchical Bayesian regression results. Regression of the parameters of the Gaussian-modelled grip force response and number of fixation shifts onto the total sum of the subjective option values from the drift diffusion model (DDM) for the high magnitude condition. Posterior distributions of the group-level parameter means. α: intercept, $\beta_1$: coefficient for grip force amplitude, $\beta_2$: coefficient for grip force centroid, $\beta_3$: coefficient for grip force width, $\beta_4$: coefficient for fixation shift. Horizontal solid lines indicate the 85% and 95% highest density interval. Vertical solid lines indicate x = 0, and vertical dashed lines indicate the lower and upper bounds of the region of practical equivalence (ROPE).
