# Supplementary material for: Motor response vigour and visual fixation patterns reflect subjective valuation during intertemporal choice
Source: PLoS Comput Biol. 2022 Jun 10;18(6):e1010096. doi: 10.1371/journal.pcbi.1010096 (PMC9187114; doi:10.1371/journal.pcbi.1010096)
Supplement: Attachment — Submitted filename: PLOS_Comp_Bio_review_11252021.docx [file pcbi.1010096.s003.docx]

Smith and Peters investigate the potential of response vigour (through gaze shifting and grip force) as an implicit measure of subjective utility during response selection of during intertemporal-choice based decision making. By combining psychophysics and modellings, the authors report a utility of response vigour in examining different aspects of decision making.

The strengths of this manuscript are two-fold. First, the authors leverage a well-thought-out and well-controlled psychophysical paradigm with rigorous cognitive models and statistical analyses, which, together, provide important findings regarding the nature of decision making. Second, the reported observations provide foundation for future experimental and computational work investigating various aspects of perceptual information processing and decision making both in health and disease.

Additionally, I appreciate the clarity with which the authors describe each component of the modelling and statistical analyses. I believe the authors’ choices for these methods are well-justified and appropriate for the question investigated. Below I have a few comments and questions that I hope the authors could discuss as they could further help place the reported findings in context of past theoretical and empirical studies.

1. The authors excluded trials where the responses were made either ‘too early’ or ‘too late’. How were the 200ms and 10s criteria were chosen? Can we think of these as ‘false alarms’ and ‘missed’ trials, accordingly? While the total number of trials that fell into these categories were small (1.81% of trials), I strongly believe it is an important conceptual idea to be considered/discussed. Specifically, the number of these trials heavily dependent on task difficulty (e.g., differential payoff/reward across conditions, time pressure), and I’m wondering if the authors could discuss how the number of ‘too early’ and ‘too late’ responses as well as task difficulty can be incorporated and interpreted in the context of the reported modelling results.
2. Along the line of the previous comment, I believe the interpretation of non-decision time (t0) is also dependent of the task setup and temporal criteria for what trials/responses to be excluded from the analyses. Can the authors expand more on their discussion of t0 in light of this?
3. I firmly believe that the findings reported in this manuscript have far reaching implications across many fields including experimental psychology, cognitive/clinical neuroscience, and computational psychiatry (as the abstract, author summary, and introduction alluded to). Yet, the discussion does not include sufficient text and references to highlight these points. One important area where I think the reported findings could be very beneficial is studies of information processing and decision making in amnesic patients as well as those aiming to examine conscious/unconscious processing related to decision making. For example, there exist several studies that reported that amnesic patients are impaired in certain processes such as statistical-learning based decision making using explicit reports (Schapiro, Gregory, Landau, McCloskey, Turk-Browne, J Cog Neuro, 2014). However, patients were found to be intact on these tasks when tested using implicit reports (e.g., Rungratsameetaweemana, Squire, Serence, PNAS, 2019). Along this line, I think the reported findings regarding the use of response vigour could greatly benefit future investigations in various clinical populations where explicit reports may not be possible/reliable.
4. Are the three sample trials for Fig 3 picked at random and are they, by and large, representative of 1) the overall modelled grip response of this subject on other trials and 2) averaged response across participants?
5. I feel that the captions of most figures could use additional explanation. For example, in Figure 3, the authors may consider labeling each of the three panels as panel a, b, c, and include text in the caption to describe what’s being plotted in blue and black etc. The tables could also benefit from more complete labels. For example, in table 4, the definitions of alpha, z, etc. are missing.
6. I would like to highlight that the authors did a great job walking readers through each of the modelling components. The modelling results were also clearly explained. However, it is unclear why the softmax rule models were models of choice in the context this task. While the DDM was well motivated in the introduction, some more background concerning why the softmax rule models were appropriate and of interested (and better candidates than other models not considered) will be very helpful.
